# Characterization of the complete mitochondrial genome and phylogenetic analysis of the Southern giant petrel (*Macronectes giganteus*, Procellariiformes: Procellariidae)

**DOI:** 10.1080/23802359.2025.2488026

**Published:** 2025-04-03

**Authors:** Jong-U Kim, Jeong-Hoon Kim

**Affiliations:** Division of Life Sciences, Korea Polar Research Institute, Incheon, South Korea

**Keywords:** *Macronectes giganteus*, mitogenome, Procellariidae, Southern giant petrel

## Abstract

The Southern giant petrel (*Macronectes giganteus* (Gmelin, 1789)) is a large seabird widely distributed in the southern oceans. In the present study, the complete mitochondrial genome of *M. giganteus* was sequenced and characterized for the first time. The mitogenome sequence was circular and 20,169 bp in length. It contains 13 protein-coding genes (PCGs) including one cis-slicing gene (ND3), 22 transfer RNA (tRNA) genes, and two ribosomal RNA (rRNA) genes. Furthermore, there was an additional copy of two tRNAs and ND6 due to a 2.9kbp duplication. The total nucleotide composition was 30.84% (A), 30.69% (C), 13.05% (G), and 25.42% (T) with an overall GC content of 43.97%. Phylogenetic analysis of all PCGs in the complete mitogenome confirmed the inclusion of *M. giganteus* within the family Procellariidae. These new mitochondrial genome data will be useful for further studies on the phylogenetics and evolutionary history of the family Procellariidae and genus *Macronectes*.

## 1. Introduction

The Southern giant petrel (*Macronectes giganteus* (Gmelin, 1789)) is a member of the family Procellariidae and the order Procellariiformes. The genus *Macronectes* comprises two extant species (*M. giganteus* and *M. halli* Mathews, 1912), and one extinct species (*M. tinae* Tennyson and Salvador, 2023) (Patterson et al. 2008). *M. giganteus* is a large seabird widely distributed in the southern oceans (Brooke 2004); it is classified as ‘least concern’ in the International Union for Conservation of Nature (IUCN) red list (IUCN 2018). The population of this species was estimated to be 38,000 pairs in the 1980s (Hunter 1985), declining to 31,000 pairs in the late 1990s (Rootes 1988). However, the global population of *M. giganteus* has increased to 54,000 breeding pairs in recent years (Birdlife International 2023). Complete mitochondrial genomes can serve as baseline information for understanding the molecular evolution and taxonomic clarification of various organisms (Sebastian et al. 2018; Kim and Kim 2021). Although genomic studies on *M. giganteus* have been performed previously (Vianna et al. 2020; Kim et al. 2021), no description of the complete mitogenome of this species or *M. halli* has been published. The present study aimed to obtain and characterize the complete mitochondrial genome of *M. giganteus* to enhance our understanding of its phylogenetic and evolutionary processes.

## 2. Materials and methods

An adult *M. giganteus* was captured by hand from a nest at the Barton Peninsula, Antarctica (62°14’3.74"S, 58°46’55.10"W), on 20 February 2011. A volume of ∼100 µl blood was collected from a major wing vein after which the bird was released to avoid negative effects on the individual. The blood sample of *M. giganteus* (proof number SGP1) was stored at the Korea Polar Research Institute, Incheon, South Korea (Dr. Jeong-Hoon Kim: jhkim94@kopri.re.kr) under proof number SGP1. The sampling locality is within the geographic range of *M. giganteus* and far outside that of *M. halli*. Total genomic DNA was extracted from the blood sample using a DNeasy Blood and Tissue kit (Qiagen, Hilden, Germany), according to the manufacturer’s instructions. The complete mitogenome was sequenced and analyzed as per previously described protocols (Kim and Kim 2021). Briefly, the 151 bp of paired-end sequencing was performed by HiSeq2500 system supplied by a commercial company (Phyzen, Seongnam, South Korea), after TruSeq DNA PCR-Free library was prepared according to the manufacturer’s instructions. The Illumina data were quality-trimmed, and used to assemble the mitochondrial genome with the CLC Assembly Cell package ver 4.2.1 (QIAGEN, Denmark). The mitochondrial genes were annotated with GeSeq (Tillich et al. 2017) and manually curated by Artemis annotation tool (Rutherford et al. 2000). The genome map of *M. giganteus* was visualized using the PMGmap tool (Zhang et al. 2024). The completeness of the mitochondrial genome was verified using sequencing depth coverage data calculated from raw data alignment to the complete genome map (Supplementary Figure S1). A phylogenetic analysis was performed with orthologous protein-coding genes of published mitogenomes of 16 relevant bird species, including 9 from Procellariiformes, 3 penguins, 3 other water birds and waterfowl as an outgroup, to assess the phylogenetic position of *M. giganteus*. Maximum likelihood (ML) phylogenies with 1,000 bootstrap replicates were obtained, and the general time reversible model with gamma distribution plus invariant sites (G + I) was applied, using the MEGA11 program (Tamura et al. 2021) after model selection with jmodeltest-2.1.10 (Darriba et al. 2012).

## 3. Results

**Figure 1.**
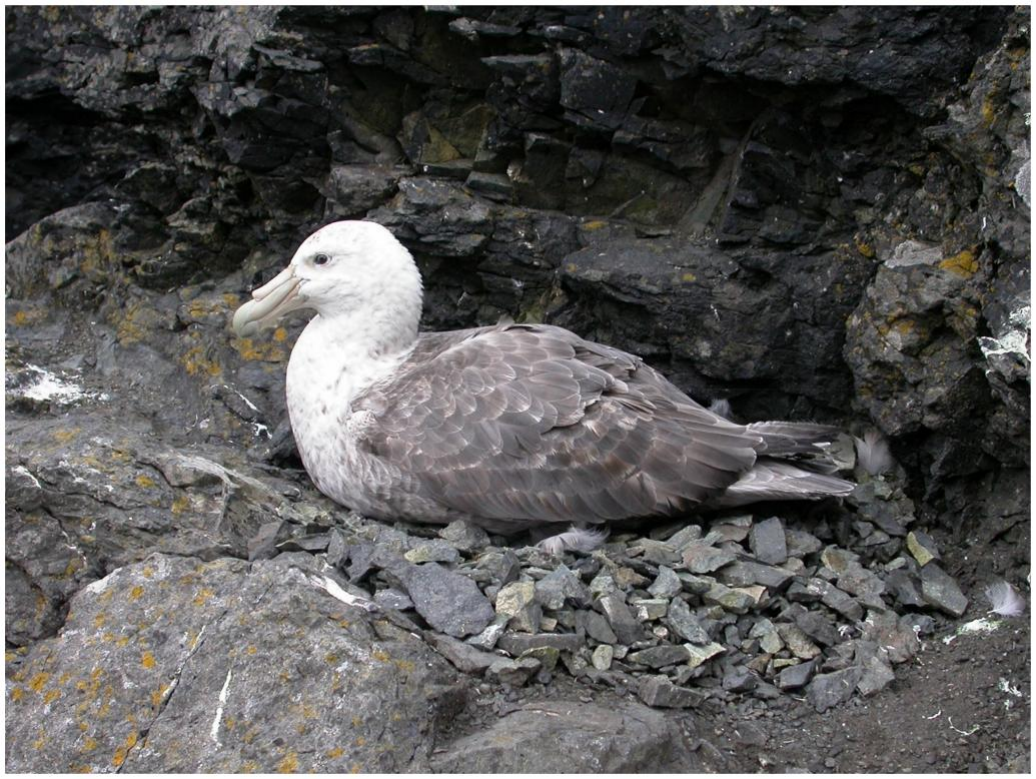
The species reference image of *Macronectes giganteus*. This photograph was taken by the corresponding author (Jeong-Hoon Kim).

**Figure 2.**
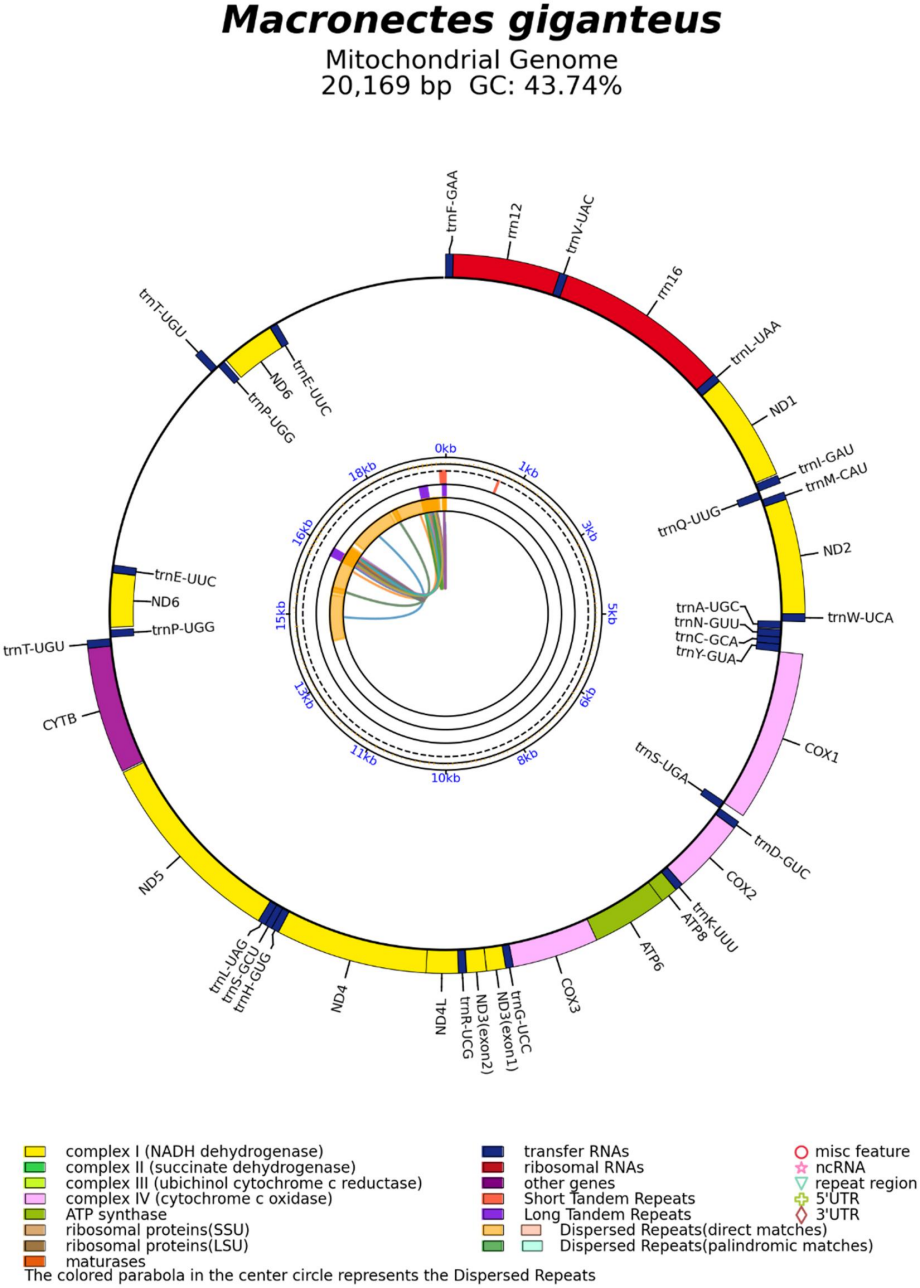
Genome map of the mitochondrial genome of *Macronectes giganteus*, consisting of 13 protein-coding, 22 transfer RNA, and two ribosomal RNA genes. Genes are shown both outside and inside the outer circle; the outside of the ring represents the positive strand, while the inside represents the negative strand.

**Figure 3.**
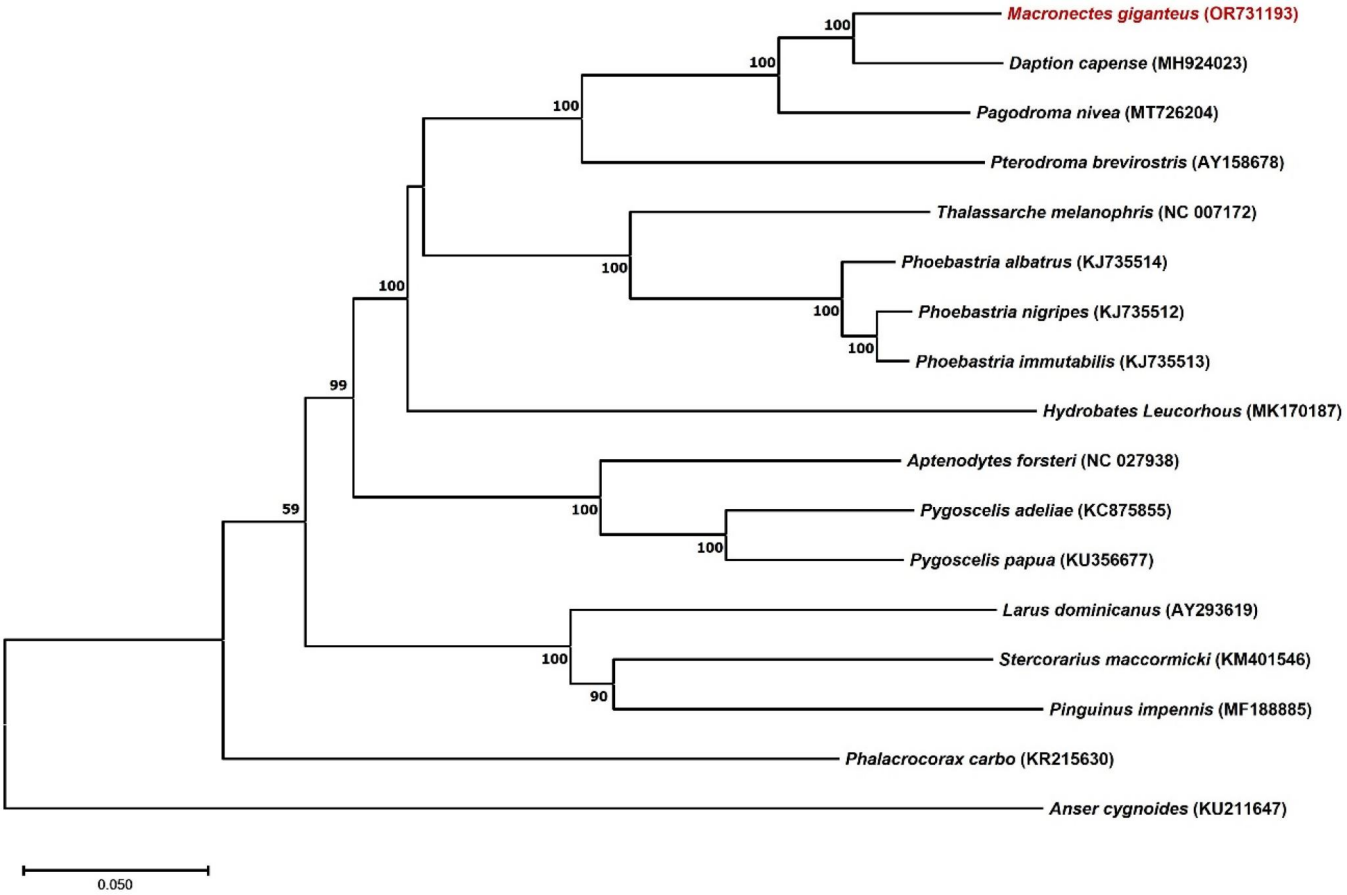
ML phylogenetic tree of *Macronectes giganteus* constructed using the published complete mitogenomes of 16 relevant bird species based on all 13 mitochondrial PCGs. The numbers on the branches indicate the ML bootstrap percentages. The species analyzed in this study is shown in red and the GenBank accession numbers of the published sequences are marked on the figure: *Aptenodytes forsteri* NC027938 (Du et al. 2019); *Pygoscelis adeliae* KC875855 (Gibb et al. 2013); *Pygoscelis Papua* KU356677 (Ramos et al. 2018); *Daption capense* MH924023 (Jung et al. 2019); *Hydrobates leucorhous* MK170187 (originally published as *Oceanodroma castro* by Antaky et al. 2019 but reidentified by Sangster and Luksenburg 2021); *Pterodroma brevirostris* AY158678 (Slack et al. 2006); *Phoebastria albatrus* KJ735514, *Phoebastria immutabilis* KJ735513, *Phoebastria nigripes* KJ735512 (Lounsberry et al. 2015); *Thalassarche melanophris* NC007172 (Slack et al. 2006); *Phalacrocorax carbo* KR215630 (Zhang et al. 2017); *Pinguinus impennis* MF188885 (Thomas et al. 2017); *Larus dominicanus* AY293619 (Slack et al. 2007); *Stercorarius maccormicki* KM401546 (Han et al. 2016); *Anser cygnoides* KU211647 (Lin et al. 2018); *Pagodroma nivea* MT726204 (Kim and Kim 2020).

The complete mitochondrial genome sequence of *M. giganteus* (Figure 1) is a circular genome that comprised 20,169 (GenBank accession no. OR731193); it has an approximately 2.9 kbp of the duplicated region (Supplementary Figure S1) and contains 13 protein-coding genes (PCGs) including one cis-slicing gene (ND3, Supplementary Figure S2), 22 transfer RNA (tRNA) genes, and two ribosomal RNA (rRNA) genes (Figure 2). The overall nucleotide base composition was 30.84% (A), 30.69% (C), 13.05% (G), and 25.42% (T), with a GC content of 43.74%. The heavy and light strands encode 28 and 9 genes, respectively. The 13 PCGs of *M. giganteus* encode 3,784 amino acids. Most PCGs started with the ATG codon, except for COI and ND1, which used the initiation codons GTG and ATT, respectively. A phylogenetic tree was constructed using all 13 PCGs to assess the phylogenetic position of *M. giganteus* with the published complete mitogenomes of 16 relevant bird species. Our phylogenetic analysis placed *M. giganteus* (OR731193) among members of the family Procellariidae. The phylogenetic analysis revealed that *Daption capense* Linnaeus, 1758 (MH924023) was the most closely related sister group with 100% bootstrap support. Further, *M. giganteus* and *D. capense* clustered with *Pagodroma nivea* Forster, 1777 (MT726204) and *Pterodroma brevirostris* Lesson, 1831 (AY158678) to form a Procellariidae clade (Figure 3).

## 4. Discussion and conclusion

To the best of our knowledge, the present study is the first to sequence and characterize the complete mitochondrial genome of *M. giganteus*. The mitogenome of *M. giganteus* is 20,169 bp long and contains 13 PCGs, 22 tRNA genes, and two rRNA genes. It has two unusual characteristics compared to other animal species; one is cis-splicing ND3 and the other is 2.9kbp of duplicated that includes two tRNAs, ND6 and the control region. The cis-splicing ND3 with an extra nucleotide has been reported in various bird and turtle species (Mindell et al. 1998) as well as in other Procellariidae species including *Pterodroma brevirostris* (AY158678.1) and *Pelecanoides urinatrix* (MN356319.1). In addition, several cases of the duplicated structure were previously reported among Procellariiformes species (Abbott et al. 2005; Eda et al. 2010; Torres et al. 2019). Thus, one-base cis-splicing ND3 and duplicated control region is characteristics of mitochondrial genome of *M. giganteus* considering previous report. The phylogenetic analysis confirmed that *M. giganteus* is among members of the Procellariidae family, which is consistent with the traditional morphological classification (Nunn and Stanley 1998; Kennedy and Page 2002, Brooke 2004). The new mitochondrial genome data from the present study will be useful for further studies on the phylogenetics and evolutionary history of the family Procellariidae and the genus *Macronectes*. Further mitogenomic studies are required to provide basic data regarding members from the genus *Macronectes* in order to better understand their relationships with other species from the family Procellariidae.

## Data Availability

The genome sequence data supporting the findings of this study are available in GenBank (https://www.ncbi.nlm.nih.gov/) under accession no. OR731193. The associated BioProject, SRA, and Bio-Sample numbers are PRJNA1031409, SRR26493500, and SAMN37935492, respectively.

## References

[CIT0001] Abbott CL, Double MC, Trueman JWH, Robinson A, Cockburn A. 2005. An unusual source of apparent mitochondrial heteroplasmy: duplicate mitochondrial control regions in *Thalassarche* albatrosses. Mol Ecol. 14(11):3605–3613. doi:10.1111/j.1365-294X.2005.02672.x. 16156827

[CIT0002] Antaky CC, Kitamura PK, Knapp IS, Toonen RJ, Price MR. 2019. The complete mitochondrial genome of the Band-rumped Storm Petrel (*Oceanodroma castro*). Mitochondrial DNA Part B. 4(1):1271–1272. doi:10.1080/23802359.2019.1591199.

[CIT0003] BirdLife International. 2023. Species factsheet: *Macronectes giganteus*. [accessed 06 December 2023]. http://datazone.birdlife.org.

[CIT0004] Brooke M. 2004. Albatrosses and Petrels Across the World. Oxford: oxford University Press.

[CIT0005] Darriba D, Taboada GL, Doallo R, Posada D. 2012. jModelTest 2: more models, new heuristics and parallel computing. Nat Methods. 9(8):772–772. doi:10.1038/nmeth.2109. PMC4594756 22847109

[CIT0006] Eda M, Kuro-O M, Higuchi H, Hasegawa H, Koike H. 2010. Mosaic gene conversion after a tandem duplication of mtDNA sequence in Diomedeidae (albatrosses). Genes Genet Syst. 85(2):129–139. doi:10.1266/ggs.85.129. 20558899

[CIT0007] Hunter S. 1985. The role of giant petrels in the Southern Ocean ecosystem. Berlin: Springer-Verlag.

[CIT0008] IUCN. 2018. The IUCN Red List of Threatened Species. [accessed 06 December 2023]. https://www.iucnredlist.org.

[CIT0009] Du J, Tian JS, Lu ZC, Zhang SJ, Song XR, Liu GY, Han JB. 2019. Identification of the complete mitochondrial genome of the king penguin *Aptenodytes patagonicus* (Sphenisciformes: Spheniscidae: *Aptenodytes*). Mitochondrial DNA B Resour. 4(2):2191–2192. doi:10.1080/23802359.2019.1623121. 33365469 PMC7687631

[CIT0010] Gibb GC, Kennedy M, Penny D. 2013. Beyond phylogeny: pelecaniform and ciconiiform birds, and long-term niche stability. Mol Phylogenet Evol. 68(2):229–238. doi:10.1016/j.ympev.2013.03.021. 23562800

[CIT0011] Han YD, Baek YS, Kim JH, Choi HG, Kim S. 2016. Complete mitochondrial genome of the South Polar Skua *Stercorarius maccormicki* (Charadriiformes, Stercorariidae) in Antarctica. Mitochondrial DNA A DNA Mapp Seq Anal. 27(3):1783–1784. doi:10.3109/19401736.2014.963811. 25268998

[CIT0012] Jung J-W, Lee H, Choi H-G, Kim J-H. 2019. Complete mitogenome of the Cape petrel *Daption capense* from Barton Peninsula, King George Island, Antarctica. Mitochondrial DNA Part B. 4(1):1704–1705. doi:10.1080/23802359.2019.1607585.

[CIT0013] Kennedy M, Page RDM. 2002. Seabird Supertrees: combining partial estimates of Procellariiform Phylogeny. The Auk. 119(1):88–108. doi:10.1093/auk/119.1.88.

[CIT0014] Kim JU, Kim JH. 2020. Complete mitochondrial genome of the snow petrel, *Pagodroma nivea*. Mitochondrial DNA B Resour. 5(3):3337–3338. doi:10.1080/23802359.2020.1820389. 33458159 PMC7783026

[CIT0015] Kim JU, Kim JH. 2021. Characterization of the Complete mitochondrial genome of the Macaroni penguin *Eudyptes chrysolophus* from Barton Peninsula, King George Island, Antarctica. Mitochondrial DNA B Resour. 6(3):972–973. doi:10.1080/23802359.2021.1888329. 33796703 PMC7995847

[CIT0016] Kim SH, Lee SJ, Jo E, Kim J, Kim JU, Kim JH, Park H, Chi YM. 2021. Genome of the Southern giant petrel assembled using third-generation DNA sequencing and linked reads reveals evolutionary traits of southern avian. Animals. 11(7):2046. doi:10.3390/ani11072046. 34359174 PMC8300169

[CIT0017] Lin Q, Jiang GT, Dai QZ. 2018. The complete Mitochondrial Genome of the *Anser cygnoides* Linnaeus, 1758 Breed Daozhou and its phylogenetic analyses. Russ J Genet, 12. 54: 1493–1497. doi:10.1134/S1022795418120086.

[CIT0018] Lounsberry ZT, Brown SK, Collins PW, Henry RW, Newsome SD, Sacks BN. 2015. Next-generation sequencing workflow for assembly of nonmodel mitogenomes exemplified with North Pacific albatrosses (*Phoebastria* spp.). Mol Ecol Resour. 15(4):893–902. doi:10.1111/1755-0998.12365. 25545584

[CIT0019] Mindell DP, Sorenson MD, Dimcheff DE. 1998. An extra nucleotide is not translated in mitochondrial ND3 of some birds and turtles. Mol Biol Evol. 15(11):1568–1571. doi:10.1093/oxfordjournals.molbev.a025884. 12572620

[CIT0020] Nunn GB, Stanley SE. 1998. Body size effects and rates of cytochrome b evolution in tube-nosed seabirds. Mol Biol Evol. 15(10):1360–1371. doi:10.1093/oxfordjournals.molbev.a025864. 9787440

[CIT0021] Patterson DL, Woehler EJ, Croxall JP, Cooper J, Poncet S, Fraser WR. 2008. Breeding distribution and population status of the Northern Giant Petrel *Macronectes halli* and Southern Giant Petrel *M. giganteus*. Mar Ornithol. 36:115–124.

[CIT0022] Ramos B, González-Acuña D, Loyola DE, Johnson WE, Parker PG, Massaro M, Dantas GPM, Miranda MD, Vianna JA. 2018. Landscape genomics: natural selection drives the evolution of mitogenome in penguins. BMC Genomics. 19(1):53. doi:10.1186/s12864-017-4424-9. 29338715 PMC5771141

[CIT0023] Rootes DM. 1988. The status of birds at Signy Island, South Orkney Islands. British Antarctic Survey Bulletin. 80:87–119.

[CIT0024] Rutherford K, Parkhill J, Crook J, Horsnell T, Rice P, Rajandream MA, Barrell B. 2000. Artemis: sequence visualization and annotation. Bioinformatics. 16(10):944–945. doi:10.1093/bioinformatics/16.10.944. 11120685

[CIT0025] Sangster G, Luksenburg JA. 2021. Sharp increase of problematic mitogenomes of birds: causes, effects and remedies. Gen Biol Evol. 13:evab210. doi:10.1093/gbe/evab210. PMC8462277 34505894

[CIT0026] Sebastian W, Sukumaran S, Zacharia PU, Gopalakrishnan A. 2018. The complete mitochondrial genome and phylogeny of Indian oil sardine, *Sardinella longiceps* and Goldstripe Sardinella, *Sardinella gibbosa* from the Indian Ocean. Conservation Genet Resour. 10(4):735–739. doi:10.1007/s12686-017-0918-7.

[CIT0027] Slack KE, Delsuc F, Mclenachan PA, Arnason U, Penny D. 2007. Resolving the root of the avian mitogenomic tree by breaking up long branches. Mol Phylogenet Evol. 42(1):1–13. doi:10.1016/j.ympev.2006.06.002. 16854605

[CIT0028] Slack KE, Jones CM, Ando T, Harrison GL, Fordyce RE, Arnason U, Penny D. 2006. Early Penguin Fossils, Plus Mitochondrial Genomes, Calibrate Avian Evolution. Mol Biol Evol. 23(6):1144–1155. doi:10.1093/molbev/msj124. 16533822

[CIT0029] Tamura K, Stecher G, Kumar S. 2021. MEGA11: molecular Evolutionary Genetics Analysis Version 11. Mol Biol Evol. 38(7):3022–3027. doi:10.1093/molbev/msab120. 33892491 PMC8233496

[CIT0030] Thomas JE, Carvalho GR, Haile J, Martin MD, Castruita JAS, Niemann J, Sinding M-HS, Sandoval-Velasco M, Rawlence NJ, Fuller E, et al. 2017. An ‛Aukward’ Tale: a genetic approach to discover the whereabouts of the last great auks. Genes. 8(6):164. doi:10.3390/genes8060164. 28617333 PMC5485528

[CIT0031] Tillich M, Lehwark P, Pellizzer T, Ulbricht-Jones ES, Fischer A, Bock R, Greiner S. 2017. GeSeq – versatile and accurate annotation of organelle genomes. Nucleic Acids Res. 45(W1):W6–W11. doi:10.1093/nar/gkx391. 28486635 PMC5570176

[CIT0032] Torres L, Welch AJ, Zanchetta C, Chesser RT, Manno M, Donnadieu C, Bretagnolle V, Pante E. 2019. Evidence for a duplicated mitochondrial region in Audubon’s shearwater based on MinION sequencing. Mitochondrial DNA A DNA Mapp Seq Anal. 30(2):256–263. doi:10.1080/24701394.2018.1484116. 30043666

[CIT0033] Vianna JA, Fernandes FAN, Frugone MJ, Figueiró HV, Pertierra LR, Noll D, Bi K, Wang-Claypool CY, Lowther A, Parker P, et al. 2020. Genome-wide analyses reveal drivers of penguin diversification. Proc Natl Acad Sci USA. 117(36):22303–22310. doi:10.1073/pnas.2006659117. 32817535 PMC7486704

[CIT0034] Zhang L, Zhang M, He S. 2017. The complete mitochondrial genome of great cormorant, *Phalacrocorax carbo* (Phalacrocorax, Phalacrocoracidae). Mitochondrial DNA A DNA Mapp Seq Anal. 28(1):1–2. doi:10.3109/19401736.2015.1053063. 27781569

[CIT0035] Zhang X, Chen H, Ni Y, Wu B, Li J, Burzyński A, Liu C. 2024. Plant mitochondrial genome map (PMGmap): A software tool for the comprehensive visualization of coding, noncoding and genome features of plant mitochondrial genomes. Mol Ecol Resour. 24(5):e13952. doi:10.1111/1755-0998.13952. 38523350

